# Identifying causes and consequences of rhizosphere microbiome heritability

**DOI:** 10.1371/journal.pbio.3002604

**Published:** 2024-04-26

**Authors:** Maggie R. Wagner

**Affiliations:** Department of Ecology & Evolutionary Biology; Kansas Biological Survey & Center for Ecological Research; University of Kansas, Lawrence, Kansas, United States of America

## Abstract

Host genotype affects microbiome composition in many plants, but the mechanisms and implications of this phenomenon are understudied. New work in *PLOS Biology* illustrates how host genotype leads to differential gene expression and fitness in bacteria of the barley rhizosphere.

Extensive research has shown that plants’ genotypes influence the composition of their associated microbial communities, or microbiomes. This phenomenon (“microbiome heritability”) was described decades ago when crop genotypes that varied in pathogen resistance or rhizobial nodulation were found to also differ in colonization by a much broader range of microbes, typically measured using plate counts [1,2]. More recently, microbiome heritability research has exploded due to advances in DNA sequencing technology. Metabarcoding—the high-throughput amplification and sequencing of barcoding genes such as the bacterial 16S rRNA gene—has revealed detailed microbiome responses to host genetic variants ranging from isogenic mutant lines to species-wide genomic diversity, in numerous plant species [3]. The observation that plants shape their microbiomes has generated new questions around the specific genetic, molecular, and physiological mechanisms underlying microbiome heritability, and around the implications for the fitness of the host plant. Several of these questions are explored in a new case study in barley published in *PLOS Biology* by Pacheco-Moreno and colleagues [4] (Fig 1).

**Fig 1:**
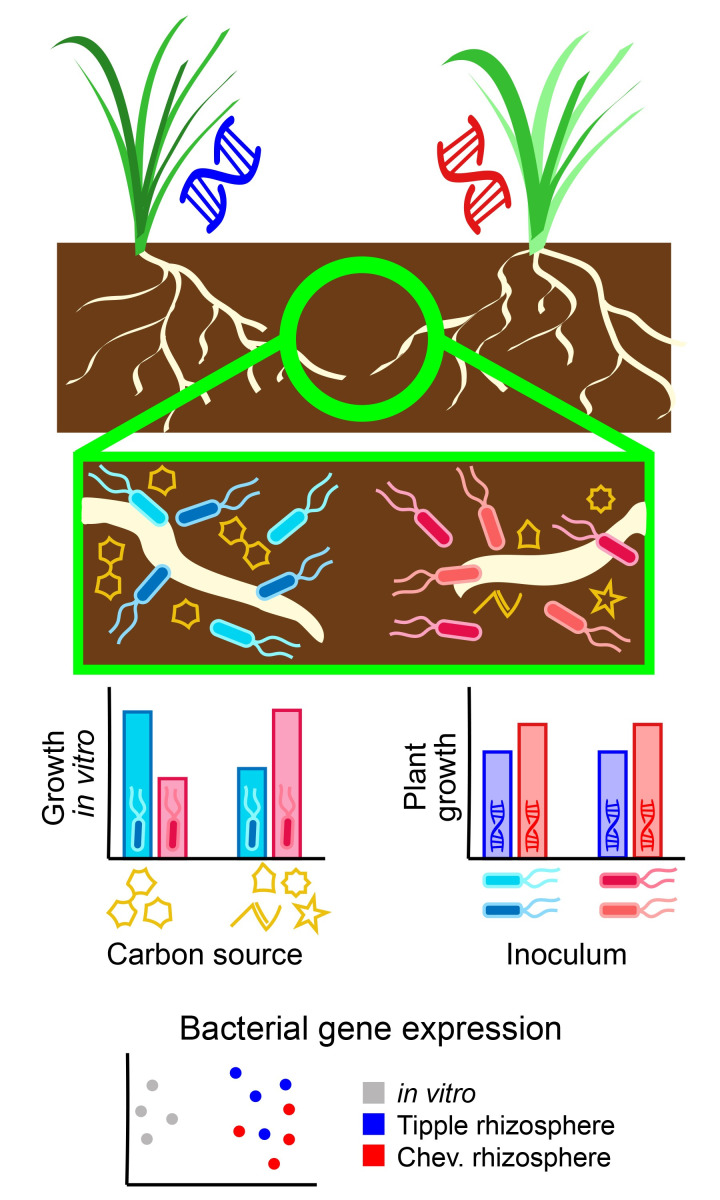
Genetic differences in root exudation have cascading effects on the fitness and gene expression of rhizosphere bacteria, but not on their plant growth-promoting abilities. Two barley cultivars (“Tipple” and “Chevallier”) differ in the chemical composition of their root exudates, particularly the types of sugars exuded into the rhizosphere. As a result, different subsets of the ambient soil microbial community become enriched in the rhizospheres of the 2 cultivars. These bacterial groups clustered phenotypically and phylogenetically by host cultivar and their host-dependent fitness differences could be recapitulated in vitro using media with carbon sources that mimicked root exudates of the 2 cultivars. Phylogenomic differences between the bacterial groups included carbon metabolism genes that, when knocked out, affected growth on one cultivar’s root exudates but not the other’s. In addition to shaping strain-level variation in microbiome composition, cultivar differences altered expression of bacterial genes related to carbon metabolism. Such host genotype effects on microbial fitness and behavior create the potential for microbiome function to evolve via selection on the host if the microbiome affects host fitness in turn. In this study, however, the 2 cultivars’ rhizosphere microbes had equivalent effects on plant growth on average. Altogether, these experiments provide an unusually complete and integrative exploration of many interacting facets of microbiome heritability.

First, how does host genotype affect fine-scale microbiome composition? Like in most microbiome heritability studies, the authors used metabarcoding to show that the rhizospheres of 2 barley genotypes—“Tipple,” a modern commercial cultivar, and “Chevallier,” an heirloom cultivar—host distinct communities of bacterial and fungal genera. Unlike in most studies, however, they also investigated strain-level microbial variation, which is challenging to detect with metabarcoding or even shotgun metagenomics [5]. To do so, they isolated >200 *Pseudomonas* colonies from barley roots, hypothesizing that the cultivars would recruit different subsets of the total *Pseudomonas* population, reflecting the strains’ adaptations to genetically variable host traits. Consistent with this hypothesis, the isolates clustered both phylogenetically and phenotypically according to the cultivar of origin. Comparative genomics identified 51 *Pseudomonas* genes that were enriched or depleted between the 2 sets of isolates, including several related to carbon metabolism. Knockouts of several of these genes impaired growth in the rhizosphere of one cultivar but not the other. Although these results from a single bacterial genus may or may not be generalizable to the rhizosphere microbiome as a whole, they demonstrate how microbiome heritability can manifest as fitness differences among strains, which go undetected by commonly used sequencing methods.

Second, what mechanisms link plant genomes to microbiomes? Potential microbiome members do not sense host genotype directly. Instead, they respond to properties of the host phenotype, which defines their potential habitat [3]. Many microbiome-relevant plant traits likely have complex genetic architectures, creating an even more complex genetic basis of microbiome composition. Despite this challenge, mutant studies and functional genomics have confirmed at least a dozen plant genes that shape microbiomes via traits including immunity, secondary chemistry, and cuticular wax [6,7]. However, most microbiome heritability studies have treated genotypes representing distinct lineages as “black boxes” full of unidentified genetic variants. In this fashion, Pacheco-Moreno and colleagues did not delve into the genetic underpinnings of barley microbiome variation. They did, however, identify a phenotypic driver of the focal cultivars’ contrasting rhizosphere microbiomes: Chevallier root exudates were more chemically diverse, but Tipple root exudates had more C6 and C12 sugars. Going further, they provided experimental evidence that these distinct exudates were a cause of the observed microbiome differentiation. Strains of *P*. *fluorescens* isolated from Tipple out-performed strains isolated from Chevallier when grown on media containing sugars that were enriched in Tipple exudates. Furthermore, knocking out 2 *P*. *fluorescens* genes required for metabolism of these different carbon sources caused host-genotype-dependent differences in bacterial relative fitness. Although the observation that root exudates shape microbial communities is not new [2,8], and although root exudation is likely not the only microbially relevant trait distinguishing these cultivars, these findings nicely illustrate the chain of causation linking host genotypes to microbiomes.

Third, how does host genotype affect microbiome behavior? Most existing studies have tested heritability of microbes’ relative abundances, a limitation of the current most accessible methods (metabarcoding). Relative abundances not only can be misleading [9], but also cannot capture community properties and emergent functions such as total microbial load and patterns of microbial activity. As a result, we know little about how host genotype influences the “behavior” of a given microbiome. Pacheco-Moreno and colleagues took steps toward addressing this deficit by comparing gene expression of a reference bacterial strain when grown in the rhizospheres of Tipple and Chevallier. Although only a handful of transcripts were differentially expressed, follow-up experiments showed that knocking out these genes led to cultivar-specific growth deficits, suggesting that *P*. *fluorescens* tailors its gene expression to maximize its competitiveness within a given host.

Finally, what are the implications of microbiome heritability for the plant? The existence of microbiome heritability raises the possibility that plant-associated microbiomes could be shaped by natural or artificial selection acting on the host. This is especially plausible given the profound effects that plant-associated microbiomes (in general) have on host health and productivity. However, to determine whether the microbiome properties affecting host fitness are the same ones that are sensitive to host genotype is challenging. Pacheco-Moreno and colleagues attempted to do so using reciprocal inoculations of each barley genotype with both full rhizosphere extracts and *Pseudomonas* isolates that were recruited by the contrasting cultivars. They found no clear evidence that either cultivar responded either positively or negatively to its own rhizosphere inoculum, compared to the other cultivar’s inoculum. In this case, because the contrasting microbiomes shaped by the 2 cultivars had no differential effect on host fitness, the necessary conditions for this “extended phenotype” to evolve by selection on the host are not satisfied [10]. Interestingly, however, all 4 inocula increased the performance of Chevallier but not Tipple, highlighting that host genotypes can also differ in their responsiveness to a given microbiome. Notably, these analyses used biomass as a proxy for fitness; measurement of true fitness components (e.g., survival or grain yield), which are more informative about host evolution, may have led to different conclusions.

Many questions remain about the causes and consequences of microbiome heritability that were not addressed in this paper. For one, although the results illustrate the general principle that root exudates strongly impact the microbial habitat in the rhizosphere, it is unclear whether genetic differences in exudate sugar content are a common mechanism of microbiome heritability in other plant species or even other barley cultivars. For another, the extent to which microbiome heritability changes over chronological time, over developmental time, and between plant organs remains understudied [3]. The consistency of microbiome heritability bears directly on whether it is feasible to breed or engineer plants that can optimize their own microbiomes. Another looming question is whether, or to what extent, the fascinating results reported by Pacheco-Moreno and colleagues apply to plants in the field, where biotic and abiotic conditions are extremely complex compared to those of these lab and greenhouse experiments. Nevertheless, this work illustrates the power of reductionist approaches to investigate previously intractable questions and inspire follow-up studies in more realistic settings. Overall, this article provides an unusually complete and integrative exploration of many interacting facets of microbiome heritability.
